# Glutathione S-transferase Mu 2-transduced mesenchymal stem cells ameliorated anti-glomerular basement membrane antibody-induced glomerulonephritis by inhibiting oxidation and inflammation

**DOI:** 10.1186/scrt408

**Published:** 2014-01-30

**Authors:** Yajuan Li, Mei Yan, Jichen Yang, Indu Raman, Yong Du, Soyoun Min, Xiangdong Fang, Chandra Mohan, Quan-Zhen Li

**Affiliations:** 1 Department of Immunology and Internal Medicine, University of Texas Southwestern Medical Center, 5323 Harry Hines Blvd, Dallas, TX 75390-8814, USA; 2 Laboratory of Disease Genomics and Individualized Medicine, Key Laboratory of Genome Sciences and Information, Beijing Institute of Genomics, Chinese Academy of Sciences, Beijing 100101, China; 3 Division of Biostatistics, Department of Clinical Sciences, University of Texas Southwestern Medical Center at Dallas, Dallas, TX 75390, USA; 4 Biomedical Engineering Department, University of Houston, Houston, TX 77204, USA; 5 Key Laboratory of Medical Genetics, Wenzhou Medical University School of Laboratory Medicine and Life Science, Wenzhou 325035, China

## Abstract

**Introduction:**

Oxidative stress is implicated in tissue inflammation, and plays an important role in the pathogenesis of immune-mediated nephritis. Using the anti-glomerular basement membrane antibody-induced glomerulonephritis (anti-GBM-GN) mouse model, we found that increased expression of glutathione S-transferase Mu 2 (GSTM2) was related to reduced renal damage caused by anti-GBM antibodies. Furthermore, mesenchymal stem cell (MSC)-based therapy has shed light on the treatment of immune-mediated kidney diseases. The aim of this study was to investigate if MSCs could be utilized as vehicles to deliver the *GSTM2* gene product into the kidney and to evaluate its potential therapeutic effect on anti-GBM-GN.

**Methods:**

The human *GSTM2* gene (*hGSTM2*) was transduced into mouse bone marrow-derived MSCs via a lentivirus vector to create a stable cell line (hGSTM2-MSC). The cultured hGSTM2-MSCs were treated with 0.5mM H_2_O_2_, and apoptotic cells were measured by terminal dUTP nick-end labeling (TUNEL) assay. The 129/svj mice, which were challenged with anti-GBM antibodies, were injected with 10^6^ hGSTM2-MSCs via the tail vein. Expression of hGSTM2 and inflammatory cytokines in the kidney was assayed by quantitative PCR and western blotting. Renal function of mice was evaluated by monitoring proteinuria and levels of blood urea nitrogen (BUN), and renal pathological changes were analyzed by histochemistry. Immunohistochemical analysis was performed to measure inflammatory cell infiltration and renal cell apoptosis.

**Results:**

MSCs transduced with *hGSTM2* exhibited similar growth and differentiation properties to MSCs. hGSTM2-MSCs persistently expressed hGSTM2 and resisted H_2_O_2_-induced apoptosis. Upon injection into 129/svj mice, hGSTM2-MSCs migrated to the kidney and expressed hGSTM2. The anti-GBM-GN mice treated with hGSTM2-MSCs exhibited reduced proteinuria and BUN (58% and 59% reduction, respectively) and ameliorated renal pathological damage, compared with control mice. Mice injected with hGSTM2-MSCs showed alleviated renal inflammatory cell infiltration and reduced expression of chemokine (C-C motif) ligand 2 (CCL2), interleukin (IL)-1β and IL-6 (53%, 46% and 52% reduction, respectively), compared with controls. Moreover, hGSTM2-MSCs increased expression of renal superoxide dismutase and catalase, which may associate with detoxifying reactive oxygen species to prevent oxidative renal damage.

**Conclusions:**

Our data suggest that the enhanced protective effect of GSTM2-transduced MSCs against anti-GBM-GN might be associated with inhibition of oxidative stress-induced renal cell apoptosis and inflammation, through over-expression of hGSTM2 in mouse kidneys.

## Introduction

Anti-glomerular basement membrane antibody-induced glomerulonephritis (anti-GBM-GN) is an autoimmune disorder in which circulating antibodies against the α-3 chain of type IV collagen bind to renal GBM and initiate an inflammatory reaction [1,2]. Anti-GBM-GN is one of the most severe forms of glomerulonephritis, characterized by crescent formation and linear glomerular deposits of IgG [3]. Patients usually present with rapidly progressive glomerulonephritis, hematuria and sub-nephrotic range proteinuria. About 40–70% of patients develop end-stage renal disease [4].

It has been reported that oxidative stress plays an important role in the pathogenesis of anti-GBM-GN, and is one of the major causes of tubulointerstitial injury [5-7]. During oxidative stress, cellular metabolism produces excessive reactive oxygen species (ROS), which modulate various physiological functions and affect innate immunity in infectious and non-infectious inflammation. ROS serve as the main products of innate immunity during the course of inflammation [8]. Overproduction of ROS, reactive nitrogen species, and reactive chlorine species by inflammatory cells in nephritis can cause further tissue damage, intensify inflammation, promote apoptosis, and accelerate the progression of nephritis [9,10]. Under physiologic conditions, there are several anti-oxidant defense mechanisms available to limit the oxidative damage. Superoxide dismutase (SOD) and catalase (CAT) are the two main anti-oxidant enzymes. SOD catalyzes the dismutation of superoxide into oxygen and hydrogen peroxide (H_2_O_2_), with the latter subsequently degraded to water and molecular oxygen by CAT or glutathione peroxidase (GPX) in the presence of reduced glutathione.

Anti-GBM-GN has been used as a model for the study of lupus nephritis because the two conditions share some common molecular pathways [11]. Our previous study showed that anti-GBM antibody challenge induced severe GN in some mouse strains such as 129/svj, DBA1, and NZW, whereas some other strains, such as B6 and BALB/c, were resistant to anti-GBM challenge, exhibiting no or very mild GN [12]. Comparing the gene expression profiles in the mouse kidneys revealed that a cluster of redox-related genes was differentially expressed between anti-GBM-resistant and anti-GBM-sensitive strains. Glutathione S-transferase Mu 2, a protein involved in detoxification of ROS, was significantly increased in anti-GBM-resistant strains (B6 and BALB/c), but decreased in anti-GBM-sensitive strains (129/svj, DBA1, and NZW), suggesting that GSTM2 may play a protective role in anti-GBM induced nephritis.

GSTM2 is a member of the glutathione S-transferase (GST) family, which participates in detoxification of ROS [13]. GSTs act as biotransformation enzymes, and exist widely in various mammalian tissues, including kidney. They play a major role in cellular anti-oxidant defense mechanisms by catalyzing the reduction of potentially harmful peroxides [14-16]. In order to elucidate the potential protective role of GSTM2 in the pathogenesis of immune-mediated nephritis, and also to explore possible therapeutic approaches using this molecule for lupus nephritis, we used genetically modified mesenchymal stem cells (MSCs) as carriers to deliver GSTM2 into the kidney of anti-GBM antibody-challenged mice, and evaluated the effects of these MSCs on anti-GBM-GN.

## Materials and methods

### Microarray and gene expression analysis

Microarray analysis on anti-GBM challenged mice has been described previously [17], and the renal gene expression profiles from five mouse strains (B6, BALB/c, 129/svj, DBA1, and NZW) were deposited in the GEO database (accession number GSE53611). The expression value of redox-related genes was extracted from the microarray data and compared for the different strains. TaqMan real-time quantitative PCR (RT-qPCR) assay was used to further confirm the expression data obtained from microarray.

### Cloning of the *GSTM2* gene into a lentiviral vector

Total RNA was extracted from human K562 cells with an RNeasy kit (Qiagen, Valencia, CA, USA) and reverse-transcribed to generate cDNA with Superscript III using random primer (Invitrogen, Carlsbad, CA, USA). Human *GSTM2* coding domain sequence (nucleotides 95 to 751, contains 219 amino acids) was amplified by PCR using the primers shown in Table 1. The hGSTM2 fragment was cloned into multiple cloning sites (MCSs) of a lentiviral vector pCDH-MSCV-MCS-EF1-GFP-Puro (System Biosciences, Mountain View, CA, USA) using *Xba*I and *Bam*HI restriction enzyme cutting sites. The resulting recombinant (pCDH-hGSTM2) DNA was amplified *in vitro,* and the sequence was verified by sequencing analysis.

**Table 1:** Primers used for PCR analysis

**Direction**	**Sequence 5′→3′**
Forward	GCTGCAGAATCCACAGCAAC
Reverse	CGCAAGGCCCTACTTGTTGC

### Transducing pCDH-hGSTM2 into MSCs

MSCs were isolated from B6 bone marrow and cultured in DMEM (Sigma-Aldrich, St. Louis, MO, USA). The cultured MSCs were grown to passage 5, and transduced with pCDH-hGSTM2 to generate an hGSTM2-MSC cell line using lentivector expression systems (System Biosciences). Briefly, pCDH-hGSTM2 was transfected into 293TN packaging cells with packaging plasmids, and the virus particles were collected after 48 hours of cell transfection. The MSCs were infected with purified pCDH-hGSTM2 virus, and cultured for 72 hours with addition of 8 μg/ml polybrene (Sigma-AldrichO), then the transduced cells were selected with 3 μg/ml puromycin. The percentages of GFP-positive cells in MSCs transduced with pCDH (vector) or pCDH-hGSTM2 were about 50% and 25%, respectively. Cell sorting by flow cytometry was used to enrich for the GFP-positive cells in both pCDH-MSCs and hGSTM2-MSCs. Expression of recombinant hGSTM2 in MSCs was assayed by RT-qPCR and western blotting. MSCs transduced with pCDH vector (pCDH-MSCs) were used as control. Only lower passage cells (before passage 10) were used for experiments.

### MSC proliferation and differentiation assay

#### ***Proliferation***

hGSTM2-MSCs were seeded in six-well plates at 10^4^ cells/well and cultured in DMEM at 37°C with 5% CO_2_ for 7 days. A set of plates was removed every 24 hours, and the cells were detached with 0.25% Trypsin-EDTA, then the total number of cells was counted under a microscope. Proliferation of untransduced MSCs and pCDH-MSCs (control cells) was also assayed. Triplicate wells were counted for each cell type at each time point.

#### ***Differentiation***

The differentiation potential of hGSTM2-MSCs was tested by assays for adipogenesis and osteogenesis. Briefly, 2 × 10^4^ cells were seeded in six-well plates. For adipogenic differentiation, the cultured cells were incubated in DMEM containing 50 μg/ml indomethacin, 0.1 μM dexamethasone, and 50 μg/ml L-ascorbic acid 2-phosphate sesquimagnesium salt hydrate (all Sigma-Aldrich). For osteogenic differentiation, the cultured cells were incubated in medium supplemented with 10 mM β-glycerophosphate disodium salt hydrate, 50 μg/ml L-ascorbic acid 2-phosphate sesquimagnesium salt hydrate, and 0.1 μM dexamethasone (all Sigma-Aldrich).

The culture medium was changed every 3 days for up to 3 weeks. The adipogenic lineage cells were fixed in 10% formalin for 30 minutes and stained with Oil Red O (Sigma-Aldrich), and the osteogenic lineage cells were also fixed with 10% formalin for 30 minutes at room temperature and stained with Alizarin red (pH 4.0; Sigma-Aldrich) for 30 minutes. The stained cells were observed under the microscope.

### Detection of H_2_O_2_-induced apoptosis *in vitro*

hGSTM2-MSCs (2 × 10^5^) were seeded in six-well plates containing poly-L-lysine-coated slides. When the cells in each well were 60 to 70% confluent, the medium in each well was removed and replaced with fresh DMEM medium with or without 0.5 mM H_2_O_2_, and the cells were cultured for a further 6 hours. Apoptotic cells were stained using an *In Situ* Cell Death Detection Kit (Roche, Indianapolis, IN) and the cell nuclei were stained with Vectashield mounting medium containing DAPI (Vector Laboratories Inc, Burlingame, CA, USA). Apoptotic cells were identified by their distinct condensed nuclei. Images were acquired using a DFC420 microscope and Application Suite Advanced Fluorescence software (Leica, Buffalo Grove, IL, USA). pCDH-MSCs and untransduced MSCs were used as controls.

### Animal experiments

Animal experiments were performed in accordance with the guidelines of the Institutional Animal Care and Use Committee of the University of Texas Southwestern Medical Center (UTSW), and were approved by the institutional ethics committee of UTSW.

To monitor the migration and localization of MSCs in mouse kidney, three 129/svj mice were injected with 10^6^ transduced MSCs via the tail vein, and renal sections were examined for GFP signal under fluorescence microscope at days 3, 5, and 7 after injection. GFP signal was also monitored in three mice injected with PBS as controls.

The protective effect of hGSTM2-MSCs was evaluated using the anti-GBM-GN mouse model as described previously [17]. Briefly, 2-month-old 129/svj mice (Jackson Laboratories, Bar Harbor, ME, USA) were randomly divided into two groups of eight mice each, and challenged with rabbit anti-mouse GBM serum [18]. Two days after anti-GBM challenge, mice were injected via the tail vein either with 10^6^ hGSTM2-MSCs (group 1) or 10^6^ pCDH-MSCs (group 2). All mice were housed at constant room temperature with free access to water and food, and were observed for 3 weeks. Blood and urine samples were collected at days 0 (pretreatment), 14, and 21 of challenge, and assayed for proteinuria and BUN. All mice were euthanized at day 21, and tissues were collected and frozen at -80°C for further analysis. Kidneys were removed and snap-frozen in liquid nitrogen, or fixed in formaldehyde for immunohistochemical analysis.

### Western blotting analysis

Expression of hGSTM2 was assayed by western blotting in cultured hGSTM2-MSCs and in the kidneys of hGSTM2-MSC-injected mice. Proteins extracted from cultured hGSTM2-MSCs or mouse kidneys were run in 4 to 10% SDS-PAGE gel, and transferred to PVDF membranes. After blocking with 5% milk, the PVDF was incubated with primary antibodies (rabbit anti-human GSTM2 antibody, 1:800 dilution, rabbit anti-GAPDH antibody, 1:500 dilution; Santa Cruz Biotechnologies, Santa Cruz, CA, USA) for 2 hours at room temperature and then washed with PBS plus 0.1% Tween 20 (PBS-T) 3 times at 10 minutes each time. The membrane was further incubated with goat anti-rabbit IgG HRP-conjugated secondary antibodies (Thermal Scientific, Rockford, IL, USA) for 1 hour followed by three washes with PBS-T. Chemiluminescence was assayed with an Amersham ECL™ advance western blotting detection kit (GE Healthcare Biosciences, Piscataway, NJ, USA). GAPDH was used as an internal control.

### Real-time RT-qPCR

Total RNA was extracted from cultured cells or mouse renal tissue using an RNeasy kit (Qiagen) and reverse-transcribed into cDNA with Superscript III using random primer (Invitrogen). RT-qPCR were carried out using TaqMan assays on a 7900 real-time PCR system (Applied Biosystems, Foster City, CA, USA) with 5 μl of 2 × TaqMan Universal PCR Master Mix, 4.5 μl of cDNA, and 0.5 μl 20 × TaqMan Gene Expression Assay Mix under the following thermal cycling conditions: 95°C for 10 minutes, followed by 40 cycles of 95°C for 15 seconds and 60°C for 1 minute. Each sample was run in triplicate. 18S rRNA (Hs03003631_g1) was used as an internal control. The TaqMan assay identification numbers of the genes measured are as follows: *Bcl2* (Mm00477631_m1), *CASP3* (Mm01195085_m1), *CAT* (Mm00437992_m1), *CCL2* (Mm00441242_m1), *CCL5* (Mm01302428_m1), *CCL7* (Mm00443113_m1), *CCR5* (Mm01216171_m1), *CD40lg* (Mm00441911_m1), *Fasl* (Mm00438864_m1), *GPX1* (Mm00656767_g1), *GSTM2* (Hs00265266_g1), *IL-1β* (Mm00434228_m1), *IL-6* (Mm99999064_m1), *NFκB1* (Mm00476361_m1), *TGF-β*_2_ (Mm00436952_m1), and *TNF* (Mm00443258_m1).

### Measurement of proteinuria and BUN

Urine samples were collected for 24 hours from all the experimental mice using metabolic cages with mice allowed free access to water. Following the relevant manufacturer’s instructions, urinary protein concentration was determined using a Coomassie Plus protein assay kit (Thermo Fisher Scientific, Rockford, IL, USA), while the serum BUN level was measured with a urea nitrogen kit (Sigma-Aldrich).

### Nitric oxide detection and antioxidase activity assay

NO level in kidney was quantified by QuantiChrom Nitric Oxide Assay Kit (BioAassay Systems, Hayward, CA, USA). Briefly, the kidney tissue was homogenized in PBS and centrifuged at 10,000 × *g* at 4°C. The supernatant was firstly deproteinized by treating with ZnSO_4_ and NaOH, then 200 μl of working reagent was added to 100 μl of sample and incubated at room temperature for 150 minutes. Finally, the optical density (OD) of each reaction was read at 540 nm. NO level was calculated based on a standard curve. SOD, and CAT activity in mouse kidney tissue were measured using relevant kits (SOD Assay Kit (Sigma-Aldrich) and Catalase Assay Kit (Cayman Chemical Company, Ann Arbor, MI, USA), following each manufacturer’s instructions.

### Kidney morphological and histological analysis

Renal tissues from mice were fixed in 10% formaldehyde, dehydrated, embedded in paraffin wax, and sectioned. Tissue sections were stained with periodic acid Schiff (PAS), and examined in a blinded manner to evaluate the degree of nephritis. The severity of GN was graded on a scale of 0 to 4. The severity of tubulointerstitial nephritis was also graded on a scale of 0 to 4, based on the extent of tubular atrophy, inflammatory infiltrates, and interstitial fibrosis, as detailed previously [12]. The mesangial matrix accumulation was also evaluated by PAS staining. Immunohistochemical staining was performed to detect mesangial cell proliferation and monocyte/macrophage infiltration with specific antibodies.

### Terminal dUTP nick-end labeling assay on kidney tissue sections

Apoptosis detection in kidney tissue sections was performed with a DeadEnd™ Colorimetric Apoptosis Detection System (Promega Corporation, Madison, WI, USA) in accordance with the manufacturer’s instructions. TUNEL-positive apoptotic cells were observed under light microscopy.

### Renal immunohistochemical analysis

Immunohistochemical staining was performed on renal sections as described previously [19]. Infiltration of lymphocytes and macrophages was assayed by staining with anti-CD3 antibody (1:200 dilution; Thermo Fisher Scientific) and anti-Iba1 antibody (1:800 dilution; Abcam, Cambridge, MA, USA), respectively. The number of CD3-positive and Iba1-positive cells was calculated in 20 high power fields (HPFs).

Levels of redox-related enzymes were also assayed by immunohistochemical staining using the following antibodies: anti-catalase (1:500; Sigma-Aldrich); anti-GPX1 (1:300) and anti-iNOS (1:200) (both Abcam). The kidney slides were stained using a biotin-free immunoenzymatic antigen detection system (Expose mouse/rabbit specific HRP/DAB IHC kit, Abcam, USA). The intensity of immunohistochemical staining signal for CAT, GPX1 and inducible nitric oxide synthase (iNOS) was evaluated using the scales described previously [20], and the positive cells were counted in 20 HPFs.

### Statistical analysis

Data were analyzed by standard statistical methods, and Student’s *t*-test was used in statistical evaluation of the data as appropriate using GraphPad Prism 5 (GraphPad Software, Inc., La Jolla, CA, USA). Group data are expressed as means ± SEM. Values of all parameters were considered significantly different at a value of *P*<0.05.

## Results

### GSTM2 expression increased in the kidney of anti-GBM-GN-resistant mice

**Figure 1:**
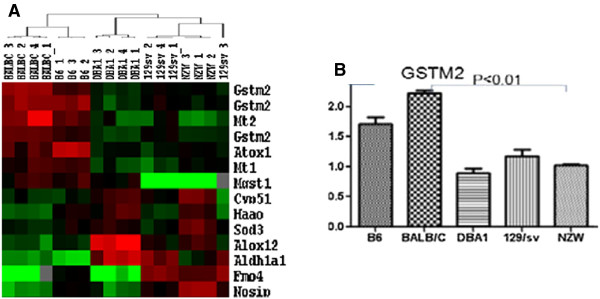
**Kidney gene expression profiling of five mice strains in an anti-glomerular basement membrane antibody-induced glomerulonephritis (anti-GBM-GN) mouse model. (A)** A group of oxidative stress-related genes that were differentially expressed between anti-GBM-GN-resistant mouse strains (B6 and BALB/c) and anti-GBM-GN-sensitive strains (DBA1, 129/svj, and NZW). The *GSTM2* gene was up-regulated in anti-GBM-GN-resistant strains compared with anti-GBM-GN-sensitive strains. **(B)** Expression of glutathione S-transferase Mu 2 (GSTM2) in different mouse strains was confirmed by reverse transcription quantitative PCR.

Our previous studies showed that different mouse strains had different sensitivity to anti-GBM-GN. NZW, DBA1, and 129/svj mice, which were sensitive to anti-GBM-GN, exhibited severe nephritis, whereas B6 and BALB/c mice, which were relatively resistant to anti-GBM-GN, had very mild nephritis [17]. Microarray gene expression analysis was performed on the five mouse strains (data are shown in the supplementary material; see Additional file 1: Figure S1; see Additional file 2: Table S1; see Additional file 3: Table S2). Comparing the gene expression profile of the redox-related genes in the kidneys of the different strains identified a group of differentially expressed genes that clearly distinguished between anti-GBM-GN-sensitive and anti-GBM-GN-resistant strains (Figure 1A). Of these genes, the *GSTM2* gene was found to be significantly increased in B6 and BALB/c mice compared with the three sensitive strains (Figure 1A). The microarray data were further confirmed by RT-qPCR assay (Figure 1B). GSTM2 is involved in detoxification of ROS [13], and was selected for further experiments in this study.

### hGSTM2-MSCs expressed hGSTM2, and maintained the differentiation property of MSCs

**Figure 2:**
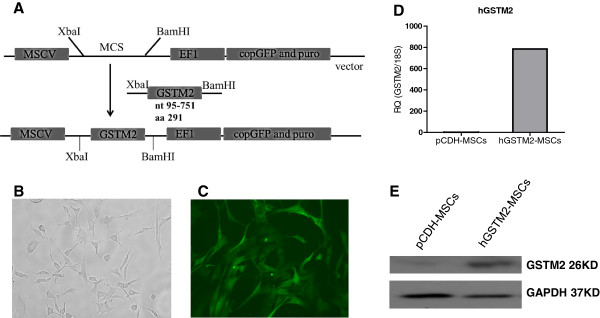
**Generation of human glutathione S-transferase Mu 2 mesenchymal stem cell (hGSTM2-MSC) cell line and detection of hGSTM2 expression. (A)** Strategy of cloning the *hGSTM2* gene into lentiviral vector pCDH-MSCV-MCS-EF1-GFP-Puro. **(B)***In vitro* cultured MSCs before transduction. **(C)** MSCs transduced with pCDH-hGSTM2. **(D, E)** Detection of hGSTM2 expression by **(D)** reverse transcription quantitative PCR and **(E)** Western blotting. hGSTM2 expression was found only in MSCs transduced with pCDH-hGSTM2 and not in MSCs transduced with pCDH vector alone.

The human *GSTM2* gene coding region was amplified by PCR, and cloned into the MCSs of a lentiviral vector, pCDH-MSCV-MCS-EF1-GFP-Puro (Figure 2A). The recombinant viral particles, pCDH-hGSTM2, were purified from package cells and transduced into MSCs. Untransduced MSCs had a fibroblast-like spindle shape (Figure 2B). hGSTM2-transduced MSCs were GFP-positive under fluorescence microscopy (Figure 2C). hGSTM2 expression was assayed by RT-qPCR and western blotting; it was found only in hGSTM2-MSCs, and not in pCDH-MSCs (Figure 2D, E).

**Figure 3:**
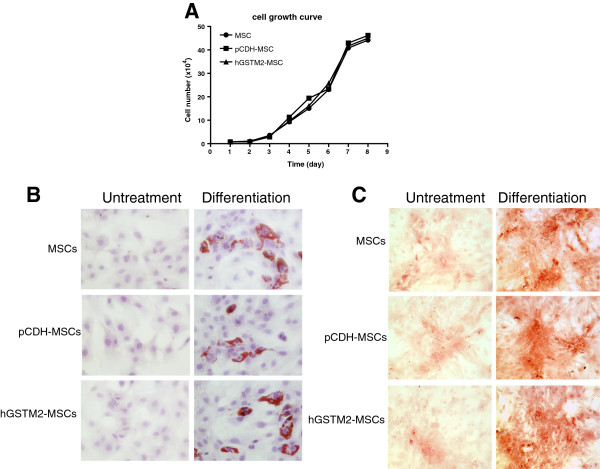
**Mesenchymal stem cells (MSCs) proliferation and differentiation characterization. (A)** Cell proliferation analysis on MSCs, pCDH-MSCs, and hGSTM2-MSCs. **(B)** The adipogenic differentiations of MSCs, pCDH-MSCs, and hGSTM2-MSCs were examined (at 21 days) with Oil Red O staining. **(C)** The osteogenic differentiations of MSCs, pCDH-MSCs, and hGSTM2-MSCs were examined (at 21 days) with Alizarin red staining (original magnification, ×200).

The cell growth curve of MSCs, pCDH-MSCs, and hGSTM2-MSCs is shown in Figure 3A; no significant difference in cell proliferation was observed between these three cell lines. The potential of MSCs to differentiate into osteocytes and adipocytes was examined by culturing the MSCs in appropriate induction medium. After 3 weeks of culture, the adipocytes were stained with Oil Red O, and adipose droplets were visible (Figure 3B). In the undifferentiated control groups, the cells maintained their fibroblast-like shape without visible formation of lipid droplets. For osteogenic differentiation, nodule-like structures, which were stained with Alizarin red, were observed after 3 weeks of induction, as shown in Figure 3C.

### hGSTM2-MSCs were more resistant to H_2_O_2_-induced apoptosis

**Figure 4:**
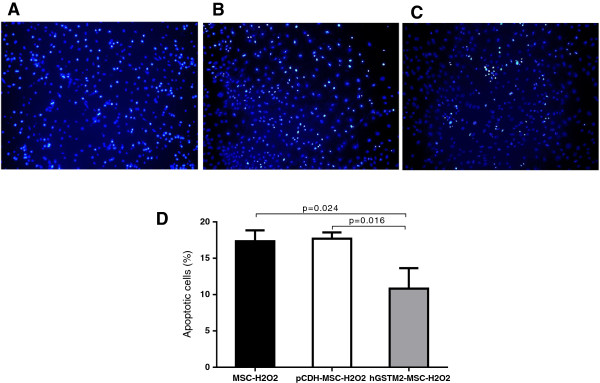
**Human glutathione S-transferase Mu 2 mesenchymal stem cells (hGSTM2-MSCs) were resistant to hydrogen peroxide-induced apoptosis. (A-C)** Detection of apoptotic cells by terminal dUTP nick-end labeling (TUNEL) staining on *in vitro* cultured MSCs treated with 0.5 mM H_2_O_2_ for 6 hours: representative images from **(A)** untransduced MSCs, **(B)** pCDH-MSCs (vector controls), and **(C)** hGSTM2-MSCs. **(D)** Quantitative analysis of apoptotic cells showed that the percentage of apoptotic cells was significantly higher for MSCs and pCDH-MSCs than for hGSTM2-MSCs (*P*<0.05).

The anti-oxidant effect of hGSTM2-MSCs was evaluated for H_2_O_2_-induced apoptosis. Treatment with 0.5 mM H_2_O_2_ for 6 hours induced a significant amount of apoptosis in MSCs and pCDH-MSCs (17% and 18% respectively) (Figure 4A, B), whereas the number of apoptotic cells in hGSTM2-MSCs was significantly reduced (11%, *P*<0.05) (Figure 4C, D), indicating that hGSTM2-MSCs were more resistant to H_2_O_2_-induced apoptosis.

### hGSTM2-MSCs localized in mouse kidney and expressed hGSTM2

**Figure 5:**
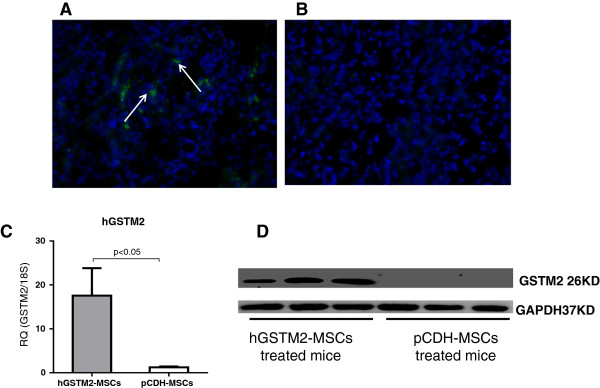
**Human glutathione S-transferase Mu 2 mesenchymal stem cells (hGSTM2-MSCs) migrated to mouse kidney and expressed hGSTM2. (A)** Assay of renal tubular and interstitial region of 129/svj mice after intravenous injection of pCDH-MSCs (vector control) or hGSTM2-MSCs. **(B)** Image of kidney from control mice receiving PBS injection. **(C-D)** Detection by **(C)** reverse transcription quantitative PCR and **(D)** Western blotting of hGSTM2 expression in the kidney of mice receiving injection of pCDH-MSCs or hGSTM2-MSCs injection (n = 3 per group).

We monitored the GFP signal of hGSTM2-MSCs; at 72 hours after tail vein injection, GFP-positive MSCs were found to have migrated and localized in the kidney tubular interstitial region of recipient mice (Figure 5A, B). Expression of hGSTM2 in mouse kidney was assayed by RT-qPCR and western blotting; it was found in the hGSTM2-MSC-injected mice, but not in the pCDH-MSC-injected mice (Figure 5C and 5D).

### hGSTM2-MSCs attenuated anti-GBM-GN

**Figure 6:**
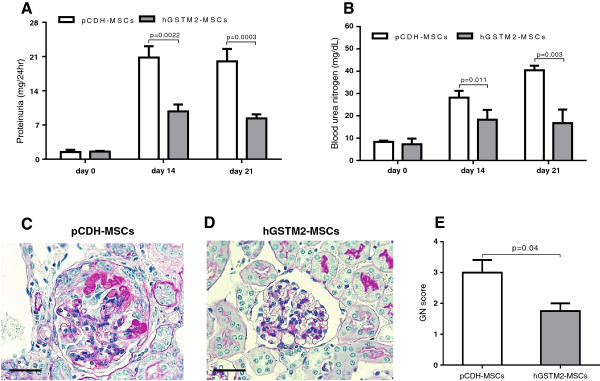
**Human glutathione S-transferase Mu 2 mesenchymal stem cells (hGSTM2-MSCs) attenuated anti-glomerular basement membrane antibody-induced glomerulonephritis (anti-GBM-GN) in 129/svj mice. (A-B)** Proteinuria and BUN were measured in 24-hour urine samples on days 0, 14, and 21 in anti-GBM-challenged 129/svj mice injected with 1 × 10^6^ pCDH-MSCs (vector controls) or the same number of hGSTM2-MSCs. **(C-D)** Renal histological images from **(C)** pCDH-MSC-injected and **(D)** hGSTM2-MSC-injected 129/svj mice challenged with anti-GBM antibody. Renal sections were stained with periodic acid Schriff (PAS). **(C)** Representative images of the kidney of pCDH-MSC-injected mice, showing some of the typical light microscopic features seen in anti-GBM-induced nephritis in 129/svj mice, including intracapillary hypercellularity, with obliteration of the capillary lumina, and tubular dilatation with casts. **(D)** Representative images of the kidneys of the anti-GBM-GN mice injected with hGSTM2-MSCs, showing mild renal histological changes. **(E)** Renal pathology score of anti-GBM-challenged 129/svj mice treated with pCDH-MSCs and hGSTM2-MSCs. Images are representative of sections from at least five mice in each study group (original magnification ×400, scale bars = 50 μm).

The protective effect of hGSTM2-MSCs on anti-GBM-GN was assessed in 129/svj mice. Before anti-GBM challenge, all mice had basal levels of proteinuria (1.52 ± 0.10 mg/24 hours) and BUN (7.80 ± 0.64 mg/dL). At day 14 after anti-GBM challenge, both pCDH-MSCs and hGSTM2-MSCs injected mice exhibited dramatically increased levels of proteinuria and BUN (Figure 6A and 6B). However, compared with pCDH-MSC mice, the hGSTM2-MSC mice showed significantly lower proteinuria (9.77 ± 0.82 mg/24 hours vs. 20.79 ± 1.35 mg/24 hours, a 53% reduction) and BUN (18.30 ± 2.19 mg/dL vs. 28.20 ± 1.50 mg/dL, a 35% reduction) (*P*<0.05). At day 21, the pCDH-MSC mice still had high levels of proteinuria (20.01 ± 1.47 mg/24 hours) and BUN (40.40 ± 1.05 mg/dL), whereas the hGSTM2-MSC mice showed further reduction in both proteinuria (8.35 ± 0.42 mg/24 hours) and BUN (16.70 ± 3.05 mg/dL), which were 59% and 58% lower than that in pCDH-MSC group (*P*<0.05) (Figure 6A, B). Histopathological analysis of kidney tissue revealed significant glomerular proliferation and crescent formation with GN score of 3.0 ± 0.4 in pCDH-MSC-injected mice (Figure 6C, E), whereas hGSTM2-MSC-injected mice exhibited significantly (*P*<0.05) less severe pathological changes, with GN score 1.8 ± 0.3 (Figure 6D, 6E). These functional manifestation and histological findings indicated that MSCs expressing hGSTM2 attenuated anti-GBM autoantibody-induced GN in 129/svj mice.

### hGSTM2-MSCs inhibited anti-GBM-induced renal cell apoptosis

**Figure 7:**
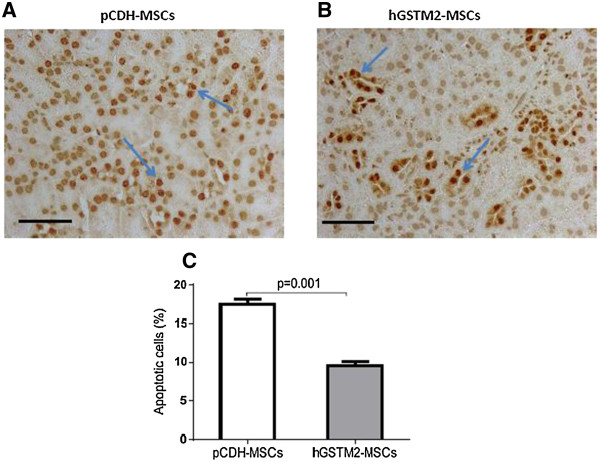
**Human glutathione S-transferase Mu 2 mesenchymal stem cells (hGSTM2-MSCs) inhibited renal cell apoptosis in anti-glomerular basement membrane antibody-induced glomerulonephritis (anti-GBM-GN) mice.** Terminal dUTP nick-end labeling (TUNEL) staining using DeadEnd™ Colorimetric Apoptosis Detection System to show apoptotic cells in renal sections of 129/svj with anti-GBM-GN injected with **(A)** pCDH-MSCs (vector control) or **(B)** hGSTM2-MSCs. Cells with brown nuclei are apoptotic cells (indicated by arrow). **(C)** Quantitative analysis of apoptotic cells expressed as a percentage of total cell number. Images were representative of sections from at least five mice in each study group (original magnification ×400, scale bars = 50 μm).

**Figure 8:**
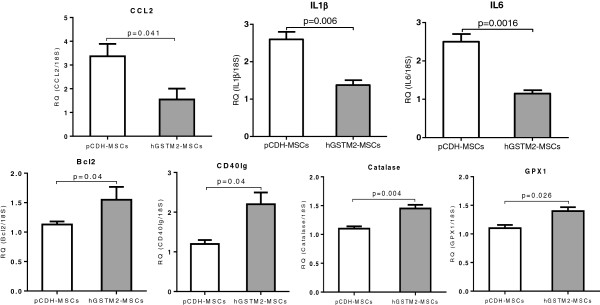
**Detection of cytokines, apoptotic factors and antioxidase genes by reverse transcription quantitative PCR (RT-qPCR) in anti-glomerular basement membrane antibody-induced glomerulonephritis (anti-GBM-GN) mice.** Expression of seven genes representing proinflammatory cytokines and apoptosis factors in the kidneys were measured by RT-qPCR on 129 mice treated with hGSTM2-MSCs or pCDH-MSCs (vector control). 18rRNA was used as internal control. Relative quantity (RQ) in each bar represents the mean of five samples. Error bars denote SEM.

Renal cell apoptosis was examined in kidney sections from mice receiving hGSTM2-MSC or pCDH-MSC transplantation. The percentage of renal apoptotic cells was significantly lower in the hGSTM2-MSC group (9.5% ± 0.5) compared with the pCDH-MSC group (17.5% ± 0.6) (*P*<0.05) (Figure 7A-C), indicating that hGSTM2-MSCs attenuated renal cell apoptosis in anti-GBM-GN. In addition, RT-qPCR showed that *Bcl2* and *CD40lg* were up-regulated in hGSTM2-MSC-treated mice (Figure 8), which may have contributed to the anti-apoptotic effect of hGSTM2-MSCs in mouse kidney.

### hGSTM2-MSCs ameliorated inflammatory cell infiltration

**Figure 9:**
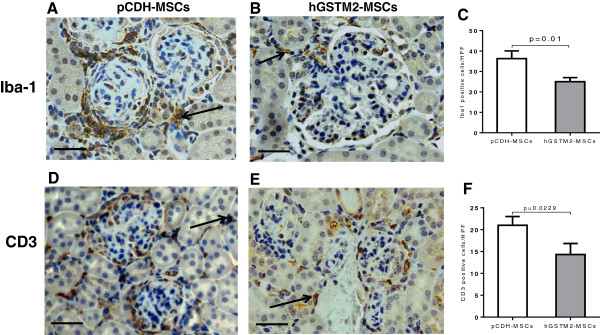
**Human glutathione S-transferase Mu 2 mesenchymal stem cells (hGSTM2-MSCs) suppressed renal inflammatory cell infiltration in mouse kidney.** 129/svj mice with anti-glomerular basement membrane antibody-induced glomerulonephritis (anti-GBM-GN) were injected with hGSTM2-MSCs or pCDH-MSCs (vector control) for 21 days, and renal inflammatory cell infiltration was analyzed by immunohistochemical analysis staining with anti-Iba1 (macrophages) and anti-CD3 (T lymphocytes). **(A, B)** Anti-Iba1 staining for macrophage infiltration in **(A)** pCDH-MSC-treated and **(B)** hGSTM2-MSC-treated mice.** (C)** Quantitative analysis of Iba1-positive cells expressed as cell numbers per high power field (*P*<0.05). **(D-E)** Anti-CD3 staining for T lymphocyte infiltration in **(D)** pCDH-MSC-treated and **(E)** hGSTM2-MSC-treated mice.** (F)** Quantitative analysis of CD3-positive cells expressed as cell numbers per high power field (*P*<0.05). Original magnification ×400, scale bars = 50 μm.

Infiltration of macrophage and T lymphocytes in the renal glomerular and interstential regions were assayed by immunohistochemical staining with anti-Iba1 and anti-CD3 antibodies. The number of infiltrated macrophages (Iba1-positive) in hGSTM2-MSC-injected mice was significantly less than in pCDH-MSC-injected control mice (25 ± 1 vs. 36 ± 2 cells per HPF, *P*<0.05) (Figure 9A-C). Similarly, the infiltrated T lymphocytes (CD3-positive) were also lower in hGSTM2-MSC-injected mice than in the pCDH-MSC-injected control mice (14 ± 1 vs. 21 ± 1 cells per HPF, *P*<0.05) (Figure 9D-F). hGSTM2-MSC treatment reduced macrophage and T lymphocyte infiltration in renal tissues by 31% and 33%, respectively, compared with pCDH-MSC-treated controls.

### hGSTM2-MSCs suppressed renal oxidative reaction

**Figure 10:**
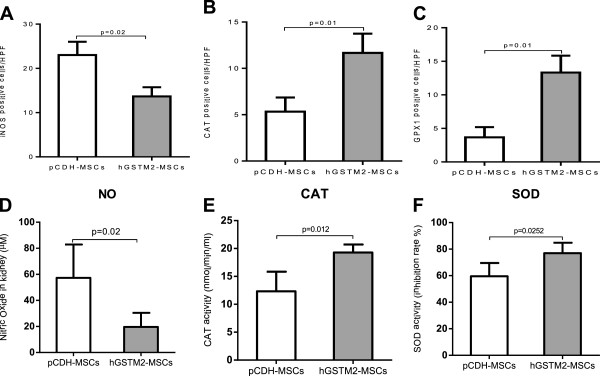
**Human glutathione S-transferase Mu 2 mesenchymal stem cells (hGSTM2-MSCs) suppressed renal oxidative reaction.** Immunohistochemical assay on cells positive for inducible nitric oxide synthase (iNOS), catalase (CAT) or glutathione peroxidase 1 (GPX1) in renal slides. **(A)** hGSTM2-MSC treatment significantly reduced the number of iNOS-positive cells compared with pCDH-MSC (vector) treatment. **(B-C)** The numbers of CAT-positive and GPX1-positive cells were significantly higher in hGSTM2-MSC-treated mice compared with control mice. **(D)** Nitric oxide (NO) level in the kidneys of mice treated with pCDH-MSCs and hGSTM2-MSCs. NO was reduced in the kidneys of hGSTM2-MSC-treated mice compared with pCDH-MSC-treated mice (*P*<0.05). **(E)** CAT and **(F)** superoxide dismutase (SOD) activities were significantly increased in the kidneys of hGSTM2-MSC-treated mice compared with pCDH-MSC-treated mice (*P*<0.05).

The effect of hGSTM2-MSCs against oxidative stress was tested by measuring the renal expression level and/or activity of oxidation-related products (iNOS, NO, CAT, GPX1 and SOD). The hGSTM2-MSC-treated mice exhibited a significantly reduced number of iNOS-positive cells (14 ± 1 cells/HPF) and lower level of NO (19.61 ± 4.85 μM), which were approximately 39% and 66% lower than that in pCDH-MSC-injected controls (23 ± 2 cells/HPF and 57.25 ± 12.79 μM, respectively, P<0.05 in Figure 10A and 10D). By contrast, the renal activity of SOD was significantly higher in hGSTM2-MSC-injected mice compared with pCDH-MSC-injected controls (76.96 ± 3.93 vs. 59.52 ± 4.48, *P*<0.05 in Figure 10F). Furthermore, the number of CAT-positive and GPX1-positive cells was much higher in hGSTM2-MSC-injected mice (12 ± 1 cells/HPF and 13 ± 1 cells/HPF), compared with pCDH-MSC-injected controls (5 ± 1 cells/HPF and 4 ± 1 cells/HPF, *P*<0.05 in Figure 10B and 10C). CAT activity was further confirmed to be significantly higher in hGSTM2-MSC-injected mice compared with pCDH-MSC-injected controls (19.28 ± 0.58 nmol/min/ml vs. 12.32 ± 1.43 nmol/min/ml, *P*<0.05 in Figure 10E).

### hGSTM2-MSCs inhibited inflammatory cytokines

The levels of inflammatory cytokines were measured by RT-qPCR. As shown in Figure 8, the chemokine *CCL2* and the cytokines *IL-1β* and *IL-6* were decreased by 53%, 46%, and 52%, respectively, in hGSTM2-MSC-injected mice compared with pCDH-MSC-injected controls (*P*<0.05). Decreased expression of several other cytokines, including *CASP3*, *CCL5*, *CCL7*, *Fasl* and *TNF*, was also found in mice transplanted with hGSTM2-MSCs, although this was not statistically significant compared with controls (data not shown).

## Discussion

It is well known that inflammatory cytokines and chemokines are involved in the pathogenesis of immune-mediated nephritis [21,22]. Immune-mediated nephritis is an autoimmune and inflammatory process involving immune cell infiltration and expression of inflammatory chemokines [23-26]. During the process of inflammation, the activated inflammatory reaction leads to the generation of oxidative radicals, such as H_2_O_2_ and ROS, and accumulation of these radicals will cause tissue damage. Excessive ROS can disturb redox status, resulting in damage to macromolecules, and can modulate the expression of a variety of immune and inflammatory molecules, further exacerbating inflammation and inducing tissue damage [27]. Further studies have shown the presence of increased oxidative stress in chronic kidney disease [28-30]. These observations implicate the relationship between ROS and chemotactic cytokine production in autoimmune disease.

In the study, using the anti-GBM-GN mouse model, we found that the increased sensitivity of 129/svj mice to anti-GBM antibody-induced renal damage may be related to impaired production of the anti-oxidant molecule GSTM2 in inflamed renal tubules. Therefore, we postulated that manipulating the expression of GSTM2 in mouse kidney might be able to combat the renal injury caused by anti-GBM antibodies.

In order to target GSTM2 expression in inflamed kidneys, we chose to use genetically modified MSCs as vehicles. MSCs have been shown to be a promising approach for targeted gene delivery because of their potential to migrate to the injured renal tissue and differentiate into intrinsic kidney cells [31,32]. Genetically modified MSCs have been used to carry kallikrein (*KLK*), *HO-1* and *PEDF* genes, which showed increased protective effect against acute ischemic kidney injury, osteoporosis, and hepatocellular carcinoma, respectively [33-35]. We recently further proved that *KLK-*transduced MSCs exhibited enhanced protective effect against anti-GBM-GN and lupus nephritis [36]. The molecular pathways underlining the protective effect of KLK in anti-GBM-GN is not yet fully understood, but the close correlation between the levels of KLK and GSTM2 in the renal tissue of anti-GBM-GN mice implies that GSTM2 might be one of the effector molecules involved in ameliorating inflammation and oxidative stress during the course of autoantibody-induced nephritis [17,18,36,37].

In the current study, we used lentiviral vector, an efficient and safe retroviral vector system to transduce the human *GSTM2* gene into mouse MSC genome. This vector carries GFP and a drug-resistant selection marker, which facilitate the cloning process. Furthermore, we used human *GSTM*2 instead of the mouse gene, to allow easy monitoring of hGSTM2 expression in mouse tissues. The stable cell line, hGSTM2-MSC, not only expressed high levels of hGSTM2, but also retained the differentiation properties of MSCs. After transplantation into 129/svj mice, the hGMTM2-MSCs migrated to the kidney and expressed high levels of hGSTM2 locally.

We tested the anti-oxidant effect of hGSTM2 *in vitro* by H_2_O_2_ stimulation assay. The data showed that hGSTM2-transduced MSCs (hGSTM2-MSCs) were more resistant to H_2_O_2_-induced apoptosis compared with vector-transduced MSCs (pCDH-MSCs). A similar effect was observed *in vivo* in mouse kidneys. The hGSTM2-MSC-injected mice exhibited much less renal cell apoptosis than the pCDH-MSC-treated controls. The anti-apoptotic effect of GSTM2 is possibly attributable to its ability to erase oxidative radicals by detoxifying ROS and superoxide [13]. It was reported that GSTM2 could inhibit oxidative damage and inflammation [13-16], and it was shown that ROS is a source of cell stress and apoptosis [8]. hGSTM2-MSC injection inhibited the activity of renal iNOS, which is a major source of reactive oxidant stress in murine models of lupus nephritis [38]. We observed that the expression of GSTM2 in hGSTM2-MSC-treated mice was accompanied by over-expression of CAT and GPX1, which are both potential scavengers of free oxidative radicals. The anti-apoptotic effects of the oxidation-resistant genes may be related to the increase in anti-apoptotic molecules such as Bcl2 and CD40lg [39,40], which were significantly up-regulated in hGSTM2-MSC-treated mouse kidneys.

The main function of GSTs involves cellular detoxification, and this detoxification process is a critical cellular defense mechanism, which can protect the cell against a series of toxic and chemical substances [41]. Although normal cells can deal with oxidative stress through intact anti-oxidative systems, GSTs and GPXs prevent the cells from further oxidative damage [42]. If the oxidative stress exceeds a defendable level or if there is a dysfunction of the cellular anti-oxidative system, the exogenous and endogenous oxidative stresses will cause oxidative damage to tissue. The ability to withstand toxic chemicals and oxidative stress is essential for the survival of all organisms, thus disruption of these redox circuits will cause oxidative stress, and even oxidative damage.

A reduction in the activity of CAT in serum was found in anti-GBM-GN, and a significant decrease in the activity of GPX was observed in patients with systemic lupus erythematosus (SLE) [19,26]. A change in redox ratio is a significant parameter for oxidative stress and such as change in favor of oxidized glutathione is observed in autoimmune disease. The levels of glutathione correlate negatively with disease activity in SLE. Low levels of glutathione have also been found in the majority of patients with SLE patients who have nephritis [26]. It has been suggested that the reduced activities of CAT and GPX in nephritis could be due to the inactivation of the enzymes by over-generation of H_2_O_2_[43]. GPX1 is one of the main cytosolic isoforms in mammalian cells, and plays a crucial role in the protection of cells against oxidative damage by H_2_O_2_[44]. GPXs, as major anti-oxidative damage enzymes, can protect cells against oxidative damage [45]. The primary role of GST is the metabolism of a broad range of ROS, and it thus plays an important role in cellular resistance against oxidative stress [46].

Oxidative stress and inflammation are two closely related pathological processes in immune-mediated tissue damage [26,47,48]. Deposition of autoantibody and/or immune complex can initiate a local immune response involving complement activation, immune cell infiltration, and inflammatory cytokine secretion. The inflammation process that recruits T cells, B cells, macrophages, and neutrophils to the inflammatory sites could generate excessive free ROS and superoxide, which will then accelerate the immunological damage in lupus nephritis. In this report, we observed that hGSTM2-MSCs suppressed the expression of inflammatory cytokines, and inhibited infiltration of lymphocytes and macrophages in renal tissues, further confirming that suppression of oxidative stress can ameliorate inflammatory damage.

Previous experiments have shown that MSCs themselves might be a therapeutic source for treatment of immune-related renal disease including lupus nephritis [49,50]. Our data indicate that transducing MSCs with therapeutic genes could significantly enhance their effect on anti-GBM-GN and lupus nephritis [36]. This study provides further evidence that MSCs could be used as vehicles to deliver potential therapeutic molecules to target tissues, and therefore may have an added beneficial effect on immune-medicated renal disease.

We plan to perform further experiments to evaluate the protective effect of hGSTM2-MSCs on lupus nephritis and diabetic nephritis, as both diseases show chronic renal inflammation. Further experiments should also be conducted to evaluate the distribution and expression of target gene in other tissues, and the long-term effect of transplanted cells, before they are trialed for treatment of human diseases.

## Conclusions

GSTM2 might be an important player in combating anti-GBM-GN. hGSTM2-MSCs suppressed the expression of inflammatory cytokines and inhibited infiltration of lymphocytes and macrophages in renal tissues, which may have contributed to the suppression of oxidative stress and amelioration of inflammatory damage. Thus, GSTM2-transduced MSCs may be a potential therapeutic agent for immune-mediated nephritis.

## Abbreviations

Bcl2: B cell leukemia/lymphoma; BUN: Blood urea nitrogen; CASP3: caspase 3; CAT: Catalase; CCL2: chemokine (C-C motif) ligand 2; CCL5: chemokine (C-C motif) ligand 5; CCL7: chemokine (C-C motif) ligand 7; CCR5: chemokine (C-C motif) receptor 5; CD40lg: CD40 ligand; DMEM: Dulbecco’s modified Eagle’s medium; Fasl: Fas ligand; GBM-GN: Glomerular basement membrane antibody-induced glomerulonephritis; GFP: Green fluorescent protein; GPX: Glutathione peroxidase; GPX1: glutathione peroxidase 1; GST: Glutathione S-transferases; GSTM2: glutathione S-transferase Mu 2; HO-1: heme oxygenase-1; HRP: horseradish peroxidase; IL-1β: interleukin 1 beta; IL-6: interleukin 6; iNOS: inducible nitric oxide synthase; KLK: kallikrein; MSC: Mesenchymal stem cells; MCSs: multiple cloning sites; NFκB1: nuclear factor of kappa light polypeptide gene enhancer in B cells 1; PAS: periodic acid Schiff; PBS-T: Phosphate-buffered saline with Tween; PEDF: pigment epithelium-derived factor; PVDF: polyvinylidene fluoride; ROS: Reactive oxygen species; SLE: Systemic lupus erythematosus; SOD: Superoxide dismutase; TGF-β2: transforming growth factor, beta 2; TNF: tumor necrosis factor; TUNEL: terminal dUTP nick-end labeling.

## Competing interests

All the authors declared no conflict of interest.

## Authors’ contributions

YL participated in the experimental design, performed the experiments, collected data, and drafted the manuscript. MY and YD performed animal experiments and sample analysis. JY performed data analysis. IR and SM performed stem cell isolation and cell culture. XF and CM participated in the design and coordination of the study. QL conceived the study, designed the experiments, and finalized the manuscript. All authors read and approved the final manuscript.

## Supplementary Material

Additional file 1: Figure S1 Expression of oxidation-related genes in the kidney cortex of five mouse strains. **(A)** Heatmap using DNA microarray of the expression value of oxidation-related genes in five mouse strains after anti-glomerular basement membrane antibody-induced glomerulonephritis (anti-GBM) challenge. Red represents up-regulation, and green represents down-regulation. **(B-F)** Quantitative PCR confirmation of the expression of five representative genes in different mice strains after anti-GBM challenge. RQ, relative quantity. Three mice were used per group. Click here for file

Additional file 2: Table S1 Transcriptomic analysis by DNA microarray of gene expression in kidneys of different mouse strains after anti-glomerular basement membrane antibody-induced glomerulonephritis (anti-GBM) challenge. Click here for file

Additional file 3: Table S2 The mRNA expression level of 15 oxidation-related genes in kidneys of five strains of mice after anti-glomerular basement membrane antibody-induced glomerulonephritis (anti-GBM) challenge. Click here for file
