# Supplementary figures and images for: Glutathione S-transferase Mu 2-transduced mesenchymal stem cells ameliorated anti-glomerular basement membrane antibody-induced glomerulonephritis by inhibiting oxidation and inflammation
Source: Stem Cell Res Ther. 2014 Jan 30;5(1):19. doi: 10.1186/scrt408 (PMC4055015; doi:10.1186/scrt408)

Figure S1

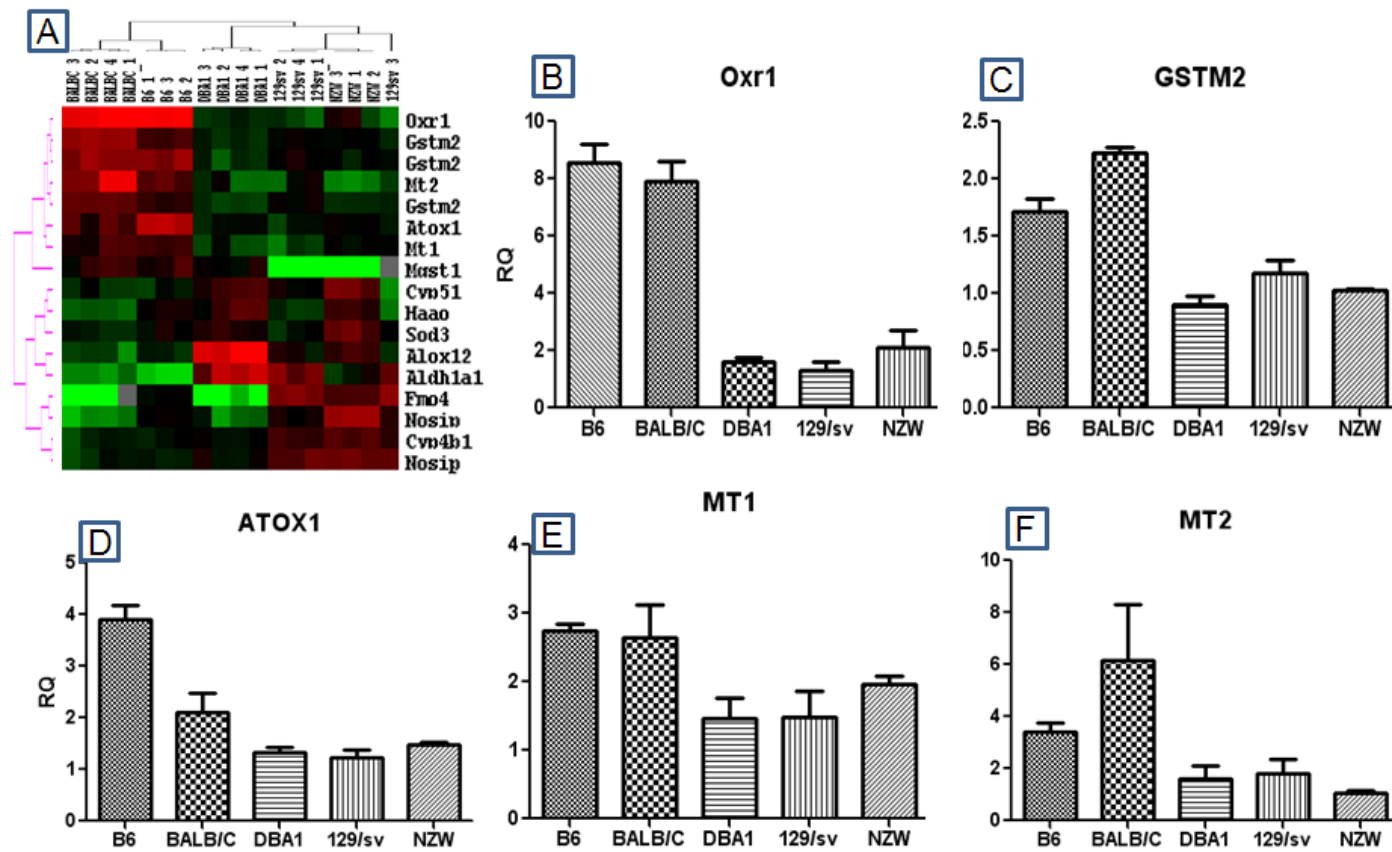

Supplement: Additional file 1: Figure S1 — Expression of oxidation-related genes in the kidney cortex of five mouse strains. (A) Heatmap using DNA microarray of the expression value of oxidation-related genes in five mouse strains after anti-glomerular basement membrane antibody-induced glomerulonephritis (anti-GBM) challenge. Red represents up-regulation, and green represents down-regulation. (B-F) Quantitative PCR confirmation of the expression of five representative genes in different mice strains after anti-GBM challenge. RQ, relative quantity. Three mice were used per group. [file scrt408-S1.pdf]
